# The Lipoprotein(a) Implementation Gap: Bridging Evidence and Clinical Practice

**DOI:** 10.31083/RCM47152

**Published:** 2026-02-25

**Authors:** Hyun Suk Yang, Seokhwan Yoon, Mina Hur

**Affiliations:** ^1^Department of Cardiovascular Medicine, Konkuk University Medical Center, 05030 Seoul, Republic of Korea; ^2^Department of Laboratory Medicine, Konkuk University School of Medicine, 05030 Seoul, Republic of Korea

**Keywords:** lipoprotein(a), implementation gap, atherosclerotic cardiovascular disease, biomarkers, mendelian randomization, RNA therapies, clinical practice guidelines

## Abstract

Lipoprotein(a) [Lp(a)] represents one of cardiovascular medicine's most profound implementation gaps: a genetically determined risk factor affecting 1.5 billion people worldwide, yet historically underutilized in clinical practice despite overwhelming evidence of its importance. This review examines the transformation of Lp(a) from an untreatable genetic burden to a promising therapeutic target through four interconnected perspectives. First, we document the implementation gap, where, despite affecting 20% of the global population, screening remains below 1%. The evolution from selective screening (2018 American College of Cardiology/American Heart Association (ACC/AHA)) to universal measurement (2024 National Lipid Association (NLA) Class I recommendation) reflects growing recognition, yet persistent barriers, including reimbursement challenges, provider knowledge gaps, and laboratory standardization issues, perpetuate underutilization. Second, we synthesize evidence establishing Lp(a)'s dual nature as both a biomarker and a causal factor. Observational studies demonstrate markedly increased cardiovascular risk with elevated Lp(a), while Mendelian randomization confirms causal relationships with coronary heart disease, large-artery stroke, peripheral artery disease, and aortic stenosis, with differential effects on stroke subtypes and non-atherosclerotic outcomes. Third, we examine the transformation from genetic determinism to pharmacological tractability. Despite 70–90% heritability, novel RNA-targeted therapies achieve unprecedented 80–95% reductions, with phase 3 cardiovascular outcome trials (completing 2026–2029) poised to determine whether dramatic Lp(a) lowering translates to clinical benefit. Finally, we provide a practical management algorithm bridging current evidence-based risk stratification with emerging therapies, stratifying patients by Lp(a) levels with corresponding interventions. The Lp(a) story exemplifies how genetic insights and technological innovation can transform immutable disease aspects into treatable conditions, offering a paradigm for precision cardiovascular medicine while highlighting the urgent need to close the gap between scientific knowledge and clinical implementation.

## 1. Introduction

Lipoprotein(a) [Lp(a)] represents one of cardiovascular medicine’s most profound implementation challenges: a genetically determined risk factor affecting 1 in 5 individuals globally, despite overwhelming evidence of Lp(a) causality and 20+ years of clinical recommendations, implementation remains remarkably poor [1]. Structurally, Lp(a) consists of a low-density lipoprotein (LDL)-like particle covalently bound to apolipoprotein(a) [apo(a)], a unique glycoprotein encoded by the *lipoprotein(a)* (*LPA*) gene on chromosome 6. However, this simple description belies the molecule’s extraordinary complexity. The apo(a) component contains multiple kringle domains with striking homology to plasminogen, positioning Lp(a) at the intersection of lipid metabolism and coagulation—a molecular chimera that defies conventional categorization [2, 3]. Table 1 summarizes the distinctive characteristics of Lp(a) compared to other apolipoprotein B-containing lipoproteins, highlighting its unique structural and metabolic properties.

**Table 1.** **Characteristics of Apo-B containing lipoprotein**.

	Lp(a)	LDL	VLDL	IDL	Chylomicrons
Structure	LDL-like with apo(a) attached to Apo-B100	One Apo-B100 per particle	One Apo-B100 per particle	One Apo-B100 per particle	Apo-B48 (truncated form)
Unique feature	Contains kringle domains similar to plasminogen	Standard Apo-B100 lipoprotein	Triglyceride-rich	Transitional lipoprotein	Largest lipoprotein
Size	25–30 nm	18–25 nm	30–80 nm	25–35 nm	75–1200 nm
Density	1.055–1.085 g/mL	1.019–1.063 g/mL	0.95–1.006 g/mL	1.006–1.019 g/mL	<0.95 g/mL
Reference range	<30 mg/dL or <75 nmol/L (optimal <50 mg/dL)	Risk-stratified targets based on ASCVD status etc. General range: <100 mg/dL (optimal) to ≥190 mg/dL (very high)	<30 mg/dL	Not routinely measured, but approximately 0–15 mg/dL	Normally absent in fasting state
Origin	Liver	VLDL catabolism	Liver	VLDL catabolism	Intestine
Half-life	3–4 days	2–3 days	1–3 hours	0.5–1 hour	Minute –hours
Genetics	Highly heritable; apo(a) size polymorphism.	Moderately heritable (40–60%); Polygenic	Polygenic	Polygenic	Minimal genetic component
	Single gene locus: *LPA* on chromosome 6q26-27; KIV-2 repeats: inverse relation to Lp(a) levels				
Metabolic regulation	Minimally affected by diet/lifestyle	Responsive to diet/statins	Very responsive to diet	Intermediate response	Directly reflects dietary fat
Atherogenic mechanisms	Foam cell formation, pro-inflammatory, pro-thrombotic, anti-fibrinolytic	Foam cell formation, oxidative modification	Contributes to IDL/LDL pool	Similar to LDL	Minimal (except remnants)
Clinical significance	Independent CVD risk factor; not targeted by standard therapies	Primary target for lipid-lowering therapy	Marker of metabolic dysregulation	Transient; less clinical focus	Relevant in hypertriglyceridemia
Measurement	Specific Lp(a) assays needed; not captured in standard lipid panel	Calculated or direct LDL-C; included in Apo-B	Reflected in triglycerides and VLDL-C	Not routinely measured	Not routinely measured
Therapeutic targets	Emerging therapies: ASO (Pelacarsen), siRNA (Olpasiran, Lepodisiran, Zerlasiran), Small molecules (Muvalaplin), Apheresis	Statins, Ezetimibe, PCSK9 inhibitors	Fibrates, Omega-3 fatty acids	Indirectly targeted by LDL therapies	Diet modification, Fibrates

Abbreviations: ASCVD, atherosclerotic cardiovascular disease; ASO, antisense oligonucleotide; CVD, cardiovascular disease; IDL, intermediate-density lipoprotein; KIV-2, kringle IV type 2; LDL, low-density lipoprotein; *LPA*, *lipoprotein(a)* gene; Lp(a), lipoprotein(a); PCSK9, proprotein convertase subtilisin/kexin type 9; siRNA, small interfering RNA; VLDL, very low-density lipoprotein; apo(a), apolipoprotein(a); LDL-C, low-density lipoprotein cholesterol; VLDL-C, very-low-density lipoprotein cholesterol.

Unlike other cardiovascular risk factors, plasma Lp(a) concentrations are predominantly (70–90%) genetically determined by variation at the *LPA* locus [4]. The apo(a) gene exhibits remarkable size polymorphism due to variable numbers of kringle IV type 2 (KIV-2) repeats, creating over 40 different isoforms in human populations [2]. This genetic diversity produces an inverse relationship between isoform size and plasma concentration: individuals inheriting smaller apo(a) variants have markedly elevated Lp(a) levels, while those with larger isoforms maintain low concentrations. Consequently, Lp(a) levels can vary over 1000-fold between individuals.

The pathophysiology of Lp(a) reveals its dual threat to vascular health. Its LDL-like component promotes atherosclerosis through cholesterol deposition and oxidative modification, while the plasminogen-like apo(a) moiety inhibits fibrinolysis and enhances thrombosis [3]. This combination of pro-atherogenic and pro-thrombotic properties distinguishes Lp(a) from other lipoproteins and may explain its particularly strong association with cardiovascular events [5]. Moreover, recent evidence suggests Lp(a) promotes vascular inflammation through oxidized phospholipid transport, adding a third dimension to its pathogenic repertoire.

However, understanding these mechanisms has not translated into clear clinical management strategies. While guidelines recognize Lp(a) as a risk enhancer, healthcare providers lack clear management strategies once results are obtained—yet this gap is rapidly closing with novel ribonucleic acid (RNA)-targeted interventions demonstrating remarkable Lp(a) reductions exceeding 80% in clinical trials [6, 7, 8, 9]. These emerging treatments have catalyzed a paradigm shift, transforming Lp(a) from an unmodifiable genetic burden to a potentially treatable condition. The implementation challenge extends beyond therapeutic nihilism: Lp(a) presents unique paradoxes as a biomarker that is simultaneously highly heritable yet now pharmacologically modifiable, causally linked to disease yet resistant to conventional therapies, and recommended for universal screening yet lacking established management pathways.

This review addresses the implementation challenges currently facing clinicians by providing: (1) a comprehensive comparison of evolving international guidelines (2018–2024) with practical laboratory standardization requirements; (2) analysis of real-world implementation barriers including reimbursement challenges and healthcare provider uncertainty; (3) synthesis of all Mendelian randomization evidence in accessible tables that distinguish atherosclerotic from non-atherosclerotic outcomes; (4) critical appraisal of therapeutic evolution from conventional to RNA-targeted approaches; and (5) a practical management algorithm for the current “bridging period” between diagnosis and RNA-targeted therapy availability. This review is designed to equip healthcare providers with actionable knowledge for managing patients with elevated Lp(a) today, while preparing for the therapeutic revolution ahead.

## 2. Current State: The Implementation Gap

### 2.1 Global Epidemiology

Lp(a) affects approximately 1.5 billion people globally. Despite being present in 20% of populations worldwide, recent data indicate that only 0.3% of adults underwent screening between 2012 and 2021 [10], creating a profound gap between epidemiological significance and clinical implementation.

Global prevalence patterns of Lp(a) levels vary significantly by ethnicity, sex, and age. Ethnic disparities in median Lp(a) levels follow a distinct pattern: individuals of African descent exhibit the highest levels (median 75 nmol/L), followed by South Asian populations (median 31 nmol/L), White individuals (median 19 nmol/L), and East Asian individuals (median 16 nmol/L) [11]. Race-specific 90th percentile thresholds demonstrate statistical significance across all ethnic groups [11]. Despite substantial ethnic differences in baseline Lp(a) levels, the relative cardiovascular risk per unit increase appears consistent across ethnic groups [11]. The distribution of apo(a) isoform size also varies by ancestry: African populations show a single peak distribution, South Asian populations demonstrate equal distribution of large and small isoforms, European populations exhibit a biphasic pattern with higher peaks on smaller isoforms, and East Asian populations display a biphasic distribution with higher peaks on larger isoforms [12].

Sex-based differences in Lp(a) levels are modest but consistent, with females demonstrating concentrations approximately 5–10% higher than males [13, 14]. Large-scale studies indicate that Lp(a) levels are generally similar between men and women before menopause but tend to be modestly higher in women afterward [11, 15, 16, 17]. Meta-analyses show that postmenopausal tibolone treatment significantly reduces Lp(a) levels by 25%, and that postmenopausal increases appear to be due to aging rather than menopause itself [18, 19].

Age-related patterns of Lp(a) levels are unique among lipoproteins: primarily genetically determined, they rise rapidly after birth, with the *LPA* gene fully expressed by age 2 and adult-like concentrations achieved by age 5 [20, 21]. After this point, Lp(a) levels remain largely unchanged throughout life, except in specific physiological or disease states [12].

### 2.2 Guideline Evolution of Standardization 

The evolution of Lp(a) recommendations from 2018 to 2024 reflects the field’s transition from uncertainty to clinical readiness (Table 2, Ref. [12, 22, 23, 24, 25]) [12, 22, 23, 26]. The 2018 American College of Cardiology/American Heart Association (ACC/AHA) guidelines adopted selective screening for high-risk individuals [23], while the 2019 European Society of Cardiology/European Atherosclerosis Society (ESC/EAS) pivotally endorsed population-wide screening, recognizing Lp(a)’s genetic determination warranted early identification regardless of therapeutic availability [24]. The 2022 EAS and 2024 National Lipid Association (NLA) guidelines marked critical inflection points by establishing detailed laboratory standards and elevating screening to a Class I recommendation, respectively [12, 22]. This progression—from selective to universal measurement with standardized protocols—coincided with promising results from phase 2 RNA-targeted intervention trials [6, 7, 9, 27].

**Table 2.** **Comparison of major clinical guidelines for lipoprotein(a) screening, risk stratification, and laboratory standards**.

Category	2018 ACC/AHA [23]	2019 ESC/EAS [24]	2022 EAS Consensus [12]	2024 NLA [22]
Screening	Selective: Measurement may be considered in individuals with family history of premature ASCVD	Universal (Class IIa): Measurement should be considered at least once in every adult’s lifetime to identify those with very high inherited Lp(a) (>430 nmol/L or >180 mg/dL)	Universal: Measure at least once in every adult’s lifetime	Universal (Class I): Measure at least once in all adults (≥18 years) for ASCVD risk assessment
			Selective: Cascade screening in families with elevated Lp(a) or premature ASCVD; consider in youth with ischemic stroke or family history	Youth <18 years (Class IIb): Screen only high-risk individuals [FH, stroke, family history of premature ASCVD, relatives with high Lp(a)]
Risk stratification: Lp(a) levels	Risk enhancer: ≥125 nmol/L (≥50 mg/dL)	• Risk enhancer: ≥125 nmol/L (≥50 mg/dL)	Continuous risk modifier:	ASCVD risk assessment (Class IIa):
			• Low risk: <75 nmol/L (<30 mg/dL)	• Low risk: <75 nmol/L (<30 mg/dL)
		• Very high risk (Class IIa): >430 nmol/L (>180 mg/dL), equivalent to FH	• Intermediate risk: between	• Intermediate risk: between
			• High risk: ≥125 nmol/L (≥50 mg/dL)	• High risk: ≥125 nmol/L (≥50 mg/dL)
Laboratory standards	No specific recommendations	No specific recommendations	Assay requirements:	Assay requirements (Class I):
			• Use isoform-insensitive immunoassays calibrated to WHO/IFCC reference material	• Use an immunochemical assay calibrated to the WHO/IFCC reference material
			• Report in nmol/L when possible	• Report in nmol/L when possible, mg/dL acceptable
			• Avoid fixed conversion factors	• Do not use fixed conversion factors
			• Do not use Lp(a)-cholesterol correction for LDL-C	• Do not use Lp(a)-cholesterol correction for LDL-C
IFCC/EFLM standards [25]	NA	NA	Not addressed	• Mandatory molar reporting (nmol/L)
				• Implement ISO 17511:2020 calibration hierarchy
				• Utilize the LC-MRM-MS reference measurement procedure for standardization
				• Ensure traceability to IFCC/WHO SRM2B
				• Regular participation in external quality assessment
Metrological best practices	NA	NA	Not explicitly addressed	• Verify apo(a) size independence of assays
				• Establish measuring range up to 600 nmol/L
				• Document calibration traceability chain
				• Implement commutability studies for reference materials
				• Maintain measurement uncertainty ≤10%

Abbreviations: ACC/AHA, American College of Cardiology/American Heart Association; EAS, European Atherosclerosis Society; EFLM, European Federation of Clinical Chemistry and Laboratory Medicine; ESC, European Society of Cardiology; FH, familial hypercholesterolemia; IFCC, International Federation of Clinical Chemistry and Laboratory Medicine; ISO, International Organization for Standardization; LC-MRM-MS, liquid chromatography-multiple reaction monitoring-mass spectrometry; NLA, National Lipid Association; SRM2B, Standard Reference Material 2B; WHO, World Health Organization. Class I = strong recommendation (benefit >>> risk); Class IIa = moderate recommendation (benefit >> risk); Class IIb = weak recommendation (benefit ≥ risk). Note: Fixed conversion factors between mg/dL and nmol/L should not be used due to apolipoprotein(a) isoform variability. The IFCC/EFLM standards represent current laboratory medicine recommendations that complement clinical society guidelines for optimal Lp(a) measurement standardization.

The standardization of Lp(a) testing is essential for reliable clinical implementation. Current standardization follows International Organization for Standardization (ISO) 17511:2020 guidelines with the International Federation of Clinical Chemistry and Laboratory Medicine (IFCC)/World Health Organization (WHO) reference material, Standard Reference Material 2B (SRM2B), serving as the primary standard [28, 29, 30]. A major advancement is the liquid chromatography-multiple reaction monitoring-mass spectrometry (LC-MRM-MS) based reference measurement procedure that measures apo(a) peptides independent of size polymorphism, offering extended measuring range up to 600 nmol/L and traceability to international standards [25]. Laboratory medicine organizations now recommend reporting in molar units (nmol/L) rather than mass concentrations (mg/dL), as mass measurements are inherently flawed due to variable apo(a) molecular weights [12]. This shift represents a paradigm change essential for accurate risk assessment across populations with different apo(a) isoform distributions. Clinically, physicians must recognize that the traditional 50 mg/dL threshold corresponds to approximately 100–125 nmol/L, with variation due to apo(a) size polymorphism, complicating risk assessment during the transition period.

Implementation of these advances faces several challenges: many laboratories still use older assays lacking proper standardization, creating significant inter-laboratory variation. The transition from mass to molar reporting requires both technical changes and clinician education for those accustomed to mg/dL thresholds. Furthermore, establishing metrological traceability chains requires coordination between manufacturers, reference laboratories, and clinical facilities—a process still in progress globally [29].

### 2.3 Barriers to Implementation

Multiple systemic barriers perpetuate the implementation gap between guidelines and clinical practice. First, reimbursement challenges create fundamental access barriers. Despite test costs of $ 20–100, Lp(a) testing faces critical reimbursement barriers globally. In the United States, Medicare explicitly does not cover Lp(a) testing, the Current Procedural Terminology (CPT) code 83695, despite covering standard lipid panels, and many private insurers classify it as ‘investigational and not medically necessary’. Similarly, in South Korea, Lp(a) measurement is not reimbursed by the National Health Insurance Service and is classified as a non-covered service. Patients must pay out-of-pocket for Lp(a) testing, which serves as a major barrier to routine screening. This creates a fundamental disconnect—recent cost-effectiveness analysis demonstrated that Lp(a) screening is cost-effective to cost-saving, with incremental cost-effectiveness ratios of 12,134 AUD/Quality-Adjusted Life Year (QALY) in Australia and actual cost savings in the UK healthcare system [31]. The Brussels International Declaration explicitly called for “systematic Lp(a) testing at least once during a person’s lifetime with full reimbursement”, representing unprecedented international consensus on the need for policy action [32].

Second, knowledge gaps among providers hinder implementation. Surveys indicate that many healthcare providers remain uncertain about how to manage patients once Lp(a) results are obtained. This uncertainty stems from the absence of specific therapies until recently and the lack of clear management algorithms. Only 4.6% of primary care providers and 14.1% of cardiologists ordered even a single Lp(a) test during the recent study periods [10].

Third, laboratory standardization issues create measurement challenges. Many laboratories still use older assays that lack proper standardization, potentially misclassifying patients as high or low risk depending on which laboratory performs the test. The transition from mass (mg/dL) to molar (nmol/L) reporting adds complexity for clinicians accustomed to traditional thresholds.

Finally, limited patient awareness reduces demand for testing. Unlike cholesterol, patients are rarely aware of Lp(a), which limits demand for testing and reduces the opportunity for cascade screening in affected families. This knowledge gap is particularly problematic given the genetic nature of elevated Lp(a) and the importance of family screening.

### 2.4 Unresolved Clinical Questions

Despite emerging consensus on universal screening and the establishment of robust metrological standards, several fundamental questions remain hotly debated in the clinical community, reflecting the complexity of translating genetic insights and analytical precision into practical care.

Reference standards and risk thresholds present a major area of ongoing debate. While current guidelines favor universal thresholds (≥125 nmol/L or ≥50 mg/dL), the substantial ethnic differences in baseline levels—with African populations having 4-fold higher median levels than East Asians—challenge this approach. This is particularly critical for sub-Saharan African populations, where applying universal thresholds would classify 40–50% as high-risk, yet most outcome data come from African Americans with different genetic admixture rather than continental African populations [11]. Despite these elevated levels, the clinical significance of Lp(a) in sub-Saharan African populations remains uncertain, though some evidence suggests that the predictive value for cardiovascular diseases may not vary sufficiently across ancestries to warrant different thresholds [11]. Critics argue that universal thresholds may lead to over-screening in some populations and under-screening in others. The question remains: Should risk stratification be ancestry-specific to account for population differences, sex-specific to address hormonal influences, or maintain universal standards for clinical simplicity? Recent genetic studies suggest that relative risk per unit increase remains consistent across ethnicities, supporting universal thresholds, but this remains an active area of investigation.

Measurement frequency and timing represent another major point of contention. While guidelines recommend “once in a lifetime” measurement based on genetic stability [12, 22, 24, 33], accumulating evidence challenges this assumption, as Lp(a) levels can fluctuate significantly during acute conditions, with certain medications, and during physiological transitions. This raises critical questions: Should Lp(a) be remeasured after major cardiovascular events? Should women be retested post-menopause? How do we account for medication-induced changes when assessing baseline risk? Current guidelines provide little guidance on these scenarios, leaving clinicians to navigate these decisions empirically.

The clinical significance of measurement variability also remains unresolved. While standardized assays exist, clinicians face unanswered questions: Is the 5–15% inter-assay variation clinically acceptable when making treatment decisions [12, 22]? Should clinicians request apo(a) isoform size testing in addition to concentration, given that smaller isoforms may confer higher risk? When patients present with results in different units (mg/dL vs nmol/L), how should clinicians interpret risk given that conversion factors vary from 2.0–2.5 depending on isoform size [12, 22]? These measurement uncertainties directly impact clinical decision-making, particularly for patients with borderline elevations where assay variability could alter risk classification.

## 3. Scientific Foundation: Biomarker vs Causal Factor

From an evolutionary perspective, Lp(a) presents a fascinating enigma. Emerging approximately 40 million years ago in primates, its conservation suggests potential adaptive advantages. Yet this molecule now confers substantial cardiovascular risk in modern humans, with levels ≥125 nmol/L (≥50 mg/dL) affecting 20% of the global population. The critical question is whether Lp(a) is merely a marker of risk or a genuine causal factor—a distinction with profound implications for intervention.

### 3.1 As a Biomarker: Robust Observational Evidence

Lp(a) has been firmly established as an independent biomarker for cardiovascular risk through decades of large-scale observational studies. The evidence base demonstrates consistent, dose-dependent associations across diverse populations and cardiovascular outcomes.

Three landmark observational studies exemplify this evidence. The Copenhagen City Heart Study (9330 participants, 10-year follow-up) demonstrated extreme Lp(a) levels predicted a 3- to 4-fold increase in myocardial infarction risk, with hazard ratios (HR) increasing stepwise from 1.2 to 2.6 across Lp(a) percentiles (trend *p* < 0.001) [34]. The Atherosclerosis Risk in Communities (ARIC) study (14,221 participants, 13.5-year follow-up) showed individuals with Lp(a) ≥30 mg/dL had 79% higher ischemic stroke risk than those <10 mg/dL, with comparable associations in African Americans and Caucasians, establishing Lp(a) as a universal biomarker across ancestries [35]. More recently, the Mass General Brigham Lp(a) Registry (16,419 participants, median 11.9-year follow-up) confirmed these associations in a large contemporary U.S. cohort, demonstrating that elevated Lp(a) independently predicts major adverse cardiovascular events in both primary and secondary prevention populations [36]. Notably, primary prevention patients with Lp(a) in the highest decile show markedly elevated cardiovascular risk, underscoring the clinical importance of identifying these individuals [36]. This recognition has led to Lp(a) being described as a biomarker whose time has come by the NLA, reflecting its maturation from research curiosity to clinical tool.

### 3.2 As a Causal Factor: Mendelian Randomization Evidence

Mendelian randomization (MR) uses genetic variants as ‘nature’s randomized trial’. Since genes are randomly assigned at conception, they’re not influenced by lifestyle or other factors that confound observational studies. If genetic variants that raise Lp(a) also increase atherosclerotic cardiovascular disease (ASCVD) risk, this strongly suggests Lp(a) causes the disease rather than just marking it. Because Lp(a) levels are largely genetically determined—primarily by *LPA* gene variants, including KIV-2 repeats and specific single nucleotide polymorphisms (SNPs)—MR is a particularly powerful tool for establishing causality [32].

Table 3 (Ref. [37, 38, 39, 40, 41, 42, 43, 44, 45, 46, 47, 48, 49, 50]) summarizes key MR studies demonstrating causal relationships between Lp(a) and ASCVD (Coronary heart disease [37, 38, 39, 40], multiple cardiovascular diseases [41, 42, 43, 44, 45, 46], ischemic stroke [41, 42, 43, 44, 45, 46], and peripheral arterial disease [50]), while Table 4 (Ref. [41, 42, 43, 44, 45, 46, 47, 48, 51, 52, 53, 54, 55, 56, 57, 58, 59, 60, 61]) presents MR evidence for non-atherosclerotic outcomes including valvular heart disease [41, 42, 43, 44, 51, 52, 53], heart failure [41, 43, 45, 46, 48, 54, 55, 56], atrial fibrillation [41, 43, 45, 46, 48, 55, 56, 57], non-ASCVD stroke [42, 45, 47], and other non-ASCVD outcomes [41, 42, 43, 46, 58, 59, 60, 61]. The MR evidence demonstrates robust causal relationships across the ASCVD outcomes. Multiple large-scale MR studies consistently show causal relationships between Lp(a) and coronary heart disease. The 2018 Burgess *et al*. study [40] notably found that a 100 mg/dL Lp(a) reduction would provide cardiovascular benefits equivalent to a 38.67 mg/dL LDL-cholesterol (LDL-C) reduction. Importantly, MR studies reveal differential effects by stroke subtype, with stronger associations for large-artery stroke (odds ratio, OR 1.20, 95% CI 1.11–1.30) [47] and null or protective effects for small-vessel stroke, suggesting distinct pathophysiological mechanisms underlying Lp(a)’s vascular effects.

**Table 3.** **Mendelian randomization studies of Lp(a) and atherosclerotic cardiovascular diseases**.

Year	Study	Population	Genetics	Outcome	MR effect (95% CI)	Key findings
2009	Kamstrup *et al*., JAMA [37]	Danish (n = 40,486; 3 studies)	KIV-2 size polymorphism (6–99 repeats)	Myocardial infarction	HR 1.22 (1.09–1.37) per doubling of Lp(a)	First large-scale MR study demonstrating Lp(a) causality.
2009	Clarke *et al*., NEJM [38]	European (PROCARDIS + replication cohorts)	rs10455872,	CHD	OR 1.70 (1.49–1.95) for rs10455872	Causal relationship confirmed; effect abolished after Lp(a) adjustment; 36% of Lp(a) variance explained.
			rs3798220		OR 1.92 (1.48–2.49) for rs3798220	
2014	Lim *et al*., PLOS Genet [39]	Finnish (n = 36,262; 3 cohorts)	*LPA* splice variants	Cardiovascular diseases	OR 0.84 (0.80–0.88) per protective allele	First demonstration of very low Lp(a) levels being protective.
2018	Burgess *et al*., JAMA Cardiol [40]	European (n = 48,333)	43 *LPA* variants	CHD	OR 0.942 (0.933–0.951) per 10 mg/dL lower Lp(a)	100 mg/dL Lp(a) reduction needed for meaningful benefit (equivalent to 38.67 mg/dL LDL-C reduction).
2016	Emdin *et al*., J Am Coll Cardiol [41]	Multiple cohorts (n = 233,000): UK Biobank (n = 112,338), 7 GWAS consortia.	4 *LPA* variants	Cardiovascular diseases	Per 1 SD lowered Lp(a):	Landmark multi-outcome study (n = 233K) establishing Lp(a) causality across CHD, stroke, and PVD per 1-SD lower Lp(a).
					CHD: OR 0.71 (0.69–0.73),	
					Stroke: OR 0.87 (0.79–0.96),	
					PVD: OR 0.69 (0.59–0.80)	
2020	Larsson *et al*., Circulation [42]	European (UK Biobank: n = 367,586)	43 *LPA* variants	Cardiovascular diseases	Per 50 mg/dL Lp(a) increase:	Largest single-population study (n = 368K) demonstrating pan-vascular effects. Establishes Lp(a) as universal vascular risk factor.
					CHD: OR 1.36 (1.32–1.40),	
					Ischemic stroke: OR 1.19 (1.12–1.25),	
					PAD: OR 1.38 (1.30–1.46),	
					AAA: OR 1.42 (1.28–1.59)	
2021	Satterfield *et al*., Circ Genom Precis Med [43]	Multi-ancestry: eMERGE, UK Biobank, MVP (804,507 EA + 103,580 AA participants)	5 *LPA* variants in EA; 5 non-overlapping variants in AA	Cardiovascular diseases	European (per 1-SD Lp(a)):	First large-scale ancestry-stratified MR study (n = 908K) showing consistent Lp(a) effects in European ancestry but weaker associations in African ancestry for CHD. PAD effects similar across ancestries, suggesting shared pathophysiology.
					CHD: OR 1.28 (1.16–1.41),	
					PAD: OR 1.22 (1.11–1.34),	
					AAA: OR 1.28 (1.27–1.40).	
					African Ancestry:	
					CHD: OR 1.11 (0.99–1.24),	
					PAD: OR 1.16 (1.01–1.33),	
					AAA: OR 1.34 (1.11–1.62)	
2021	Guertin *et al*., Circ Genom Precis Med [44]	UK Biobank (n = 408,403, European ancestry) + EPIC-Norfolk validation (n = 18,721)	27 *LPA* variants (circulating Lp(a)) + 80 *LPA* variants (hepatic *LPA* gene expression)	Cardiovascular diseases	Lp(a) (top vs bottom tertile):	First comprehensive sex-specific MR study of Lp(a); higher Lp(a) increases CHD risk in both sexes; for ischemic stroke, similar effect sizes but only significant in men.
					CHD: OR 1.32 (1.27–1.36) men, OR 1.20 (1.15–1.25) women.	
					ischemic stroke: OR 1.11 (1.02–1.22) men, OR 1.12 (0.99–1.27) women	
2022	Wang *et al*., Eur J Med Res [45]	European (n = 377,590)	18 *LPA* variants	Cardiovascular diseases	CHD: OR 1.003 (1.001–1.004),	Comprehensive MR study (n = 377K) confirming modest causal effects on specific cardiovascular outcomes. Stroke subtype specificity suggests distinct pathophysiological mechanisms.
					Large artery stroke: OR 1.003 (1.002–1.004),	
					Ischemic stroke: OR 1.001 (1.000–1.001)	
					AA: OR 1.005 (1.001–1.010)	
2025	Wang *et al*., Int J Epidemiol [46]	European (UK Biobank: n = 385,917)	43 *LPA* variants	Cardiovascular diseases	Per 50 mg/dL Lp(a) decrease:	First factorial MR study demonstrating strongest Lp(a) effect on PAD. Generally additive with LDL-C lowering, suggesting independent therapeutic targets.
					CHD: HR 0.79 (0.79–0.79),	
					Ischemic stroke: HR 0.92 (0.88, 0.96),	
					PAD: HR 0.73 (0.73–0.73)	
2019	Pan *et al*., Stroke [47]	European (MEGASTROKE consortium, n = 446,696)	9 *LPA* variants	Ischemic stroke	Per 1-SD log Lp(a) increase:	First comprehensive MR study of stroke subtypes; revealed “double-edged sword” effect - increased large artery but decreased small vessel stroke risk.
					Large-artery stroke: OR 1.20 (1.11–1.30),	
					Ischemic stroke: OR 1.02 (1.002–1.04)	
2021	Xia *et al*., Lipids Health Dis [48]	Han Chinese CHCN-BTH cohort (n = 1256)	13 SNPs from multiple genes	Ischemic stroke	OR 1.01 (0.981–1.043)	Inverse Lp(a) associations: reduced atrial fibrillation (OR 0.94), arrhythmia (OR 0.96), and cardiac remodeling, ethnic differences and lower baseline Lp(a) in Chinese population.
2023	Huang *et al*., Front Aging Neurosci [49]	Multiple cohorts: MEGASTROKE (n = 520,000), UK Biobank (n = 255,286)	8 *LPA* variants	Ischemic stroke	Total stroke: OR 1.003 (1.001–1.006),	Updated MEGASTROKE validation (n = 520K) confirming Lp(a) causality for large-artery stroke. Establishes stroke subtype specificity across multiple populations.
					Large-artery stroke: OR 1.012 (1.004–1.019),	
					Ischemic stroke: OR 1.004 (1.001–1.007)	
2023	Thomas *et al*., J Am Coll Cardiol [50]	Danish Copenhagen General Population Study (n = 108,146)	*LPA* KIV-2 repeats, rs3798220, rs10455872	PAD, AAA	PAD: RR 1.39 (1.24–1.56)	Definitive MR evidence (n = 108K): 3-fold PAD risk, 2.2-fold AAA risk with extreme Lp(a) levels. Strong genetic causality confirmed.
					per 50 mg/dL Lp(a)	
					AAA: RR 1.21 (1.01–1.44)	

Abbreviations: AA, African ancestry; AAA, abdominal aortic aneurysm; CHD, coronary heart disease; CHCN-BTH, Cohort Study on Chronic Disease of Communities Natural Population in Beijing, Tianjin and Hebei; CI, confidence interval; EA, European ancestry; eMERGE, Electronic Medical Records and Genomics; HR, hazard ratio; KIV-2, kringle IV type 2; LDL-C, low-density lipoprotein cholesterol; Lp(a), lipoprotein(a); MEGASTROKE, Multiancestry Genome-wide Association Study of Stroke; MR, Mendelian randomization; MVP, Million Veteran Program; OR, odds ratio; PAD, peripheral artery disease (also known as peripheral vascular disease, PVD); PROCARDIS, Precocious Coronary Artery Disease; RR, relative risk; SD, standard deviation; SNPs, single nucleotide polymorphisms; EPIC-Norfolk, European Prospective Investigation into Cancer and Nutrition-Norfolk.

**Table 4.** **Mendelian randomization studies of Lp(a) and non-atherosclerotic outcomes**.

**Table 4A.** **Valvular heart disease**.

Year	Study	Population	Genetics	Outcome	Effect estimate (95% CI)^a^
2013	Thanassoulis *et al*., NEJM [51]	European	rs10455872	AVC	OR 1.62 (1.27–2.06) per log-unit
2014	Arsenault *et al*., Cir Cardiovasc Genet [52]	EPIC-Norfolk + MHIB	rs10455872	AS	HR 1.95 (1.34–1.60)
2014	Kamstrup *et al*., J Am Coll Cardiol [53]	Danish cohorts	3 *LPA* variants	AS	RR 1.6 (1.2–2.1) per 10-fold Lp(a)
2016	Emdin *et al*., J Am Coll Cardiol [41]	UK Biobank	4 *LPA* variants	AS	OR 0.63 (0.47–0.83) per SD decrease
2020	Larsson *et al*., Circulation [42]	UK Biobank	43 *LPA* variants	AS, AR, MR	AS only: OR 1.74 (1.59–1.89); AS with AR: OR 1.50 (1.23–1.93), AR: OR 1.23 (1.06–1.43); MR: OR 1.11 (1.03–1.20) per 50 mg/dL
2021	Guertin *et al*., Circ Genom Precis Med [44]	UK Biobank	27 + 80 *LPA* variants	CAVS	Men: OR 1.43 (1.27–1.60); Women: OR 1.45 (1.24–1.70)
2021	Satterfield *et al*., Circ Genom Precis Med [43]	Multi-ancestry: European, African	5 *LPA* variants	AVD, MVD	European: AVD OR 1.34 (1.10–1.62), MVD OR 1.18 (1.09–1.27); African: AVD OR 1.08 (0.94–1.25), MVD OR 1.02 (0.89–1.16)

**Table 4B.** **Heart failure**.

Year	Study	Population	Genetics	Effect estimate (95% CI)^a^
2016	Emdin *et al*., J Am Coll Cardiol [41]	Multiple cohorts	4 *LPA* variants	OR 0.83 (0.73–0.94) per SD decrease
2016	Kamstrup and Nordestgaard, JACC Heart Fail [54]	Danish cohorts	3 *LPA* variants	OR 1.18 (1.04–1.34) per 10-fold Lp(a)^b^
2021	Jiang *et al*., Nutr Metab Cardiovasc Dis [55]	European	30 SNPs	OR 1.000 (0.999–1.000)
2021	Xia *et al*., Lipids Health Dis [48]	Han Chinese	13 SNPs	OR 0.99 (0.95–1.04)
2021	Satterfield *et al*., Circ Genom Precis Med [43]	Multi-ancestry: European, African	5 *LPA* variants	European: OR 1.12 (1.05–1.19); African: OR 1.02 (0.95–1.10)
2022	Wang *et al*., Eur J Med Res [45]	European	18 *LPA* variants	OR 0.999 (0.997–1.002)
2024	Singh *et al*., Curr Probl Cardiol [56]	Meta-analysis: 7 MR studies (multi-ancestry)	5–30 SNPs	OR 1.064 (1.043–1.086), *I*^2^ = 97.6%
2025	Wang *et al*., Int J Epidemiol [46]	UK Biobank	43 *LPA* variants	OR 0.89 (0.86–0.91) per 50 mg/dL decrease

**Table 4C.** **Atrial fibrillation**.

Year	Study	Population	Genetics	Effect estimate (95% CI)^a^
2016	Emdin *et al*., J Am Coll Cardiol [41]	Multiple cohorts	4 *LPA* variants	OR 0.95 (0.86–1.04) per SD decrease
2021	Jiang *et al*., Nutr Metab Cardiovasc Dis [55]	European	30 SNPs	OR 1.001 (1.000–1.003) per unit
2021	Xia *et al*., Lipids Health Dis [48]	Han Chinese	13 SNPs	OR 0.94 (0.90–0.99)
2021	Satterfield *et al*., Circ Genom Precis Med [43]	Multi-ancestry	5 *LPA* variants	European: OR 1.06 (1.04–1.09); African: OR 1.06 (0.99–1.14)
2022	Mohammadi-Shemirani *et al*., J Am Coll Cardiol [57]	Multiple cohorts	15 *LPA* variants	OR 1.03 (1.02–1.05) UK Biobank, OR 1.03 (1.02–1.05) Nielsen, OR 1.08 (1.04–1.12) FinnGen, per 50 nmol/L
2022	Wang *et al*., Eur J Med Res [45]	European	18 *LPA* variants	OR 1.001 (1.000–1.002), *p* = 0.097
2024	Singh *et al*., Curr Probl Cardiol [56]	Meta-analysis: 5 MR studies (multi-ancestry)	5–30 SNPs	OR 1.024 (1.007–1.042), *I*^2^ = 87.8%; European: OR 1.023 (1.007–1.040); Chinese: OR 0.940 (0.893–0.990)
2025	Wang *et al*., Int J Epidemiol [46]	UK Biobank	43 *LPA* variants	OR 0.94 (0.92–0.96) per 50 mg/dL decrease

**Table 4D.** **Non-ASCVD stroke**.

Year	Study	Population	Genetics	Outcome	Effect estimate (95% CI)^a^
2019	Pan *et al*., Stroke [47]	European	9 *LPA* variants	SVS, CES	SVS: OR 0.92 (0.88–0.97); CES: NS
2020	Larsson *et al*., Circulation [42]	UK Biobank	43 *LPA* variants	ICH, SAH	ICH: OR 1.00 (0.87–1.14), SAH: 1.09 (0.97–1.22)
2022	Wang *et al*., Eur J Med Res [45]	European	18 *LPA* variants	LS, SVS	LS: OR 1.000 (1.000–1.001); SVS: OR 0.999 (0.998–1.001)

**Table 4E.** **Other non-ASCVD outcomes**.

Year	Study	Population	Genetics	Outcome	Effect estimate (95% CI)^a^
2016	Emdin *et al*., J Am Coll Cardiol [41]	Multiple cohorts	4 *LPA* variants	VTE, CKD, T2D	VTE: OR 0.99 (0.91–1.07); CKD: OR 0.91 (0.83–1.00); T2D: OR 0.97 (0.92–1.03) per SD decrease
2020	Larsson *et al*., Circulation [42]	UK Biobank	43 *LPA* variants	DVT, PE, AD	DVT: OR 1.00 (0.96–1.04), PE: OR 1.01 (0.95–1.07), AD: OR 0.96 (0.84–1.11)
2021	Satterfield *et al*., Circ Genom Precis Med [43]	Multi-ancestry	5 *LPA* variants	CKD	European: OR 1.07 (1.01–1.14); African: OR 1.05 (0.99–1.12)
2022	Larsson *et al*., Metabolism [58]	UK Biobank, FinnGen	2 *LPA* variants	Multiple^c^	Anemia: OR* 1.07–1.08; T2D: OR 1.07 (1.03–1.10); Renal failure: OR 1.08 (1.04–1.11) per 50 mg/dL
2024	Goławski *et al*., Arch Med Sci [59]	UK Biobank + FinnGen	23 *LPA* variations	T2D	OR 1.008 (0.991–1.026)
2024	Yeung *et al*., Atheroscler Plus [60]	UK Biobank + Diamante	37 *LPA* variants	T2D	OR 1.02 (0.99–1.04)
2025	Wang *et al*., Int J Epidemiol [46]	UK Biobank	43 *LPA* variants	VTE	HR 0.95 (0.92–0.99) per 50 mg/dL decrease
2025	Ti *et al*., Sci Rep [61]	European consortia	4–77 *LPA* variants	IMID^d^	All NS (*p * > 0.05)

^a^All estimates per unit increase in Lp(a) unless otherwise specified; ^b^Excluding prior MI/AS: OR 1.08 (0.92–1.27); ^c^Phenome-wide analysis of 1081 phenotypes; ^d^Includes Crohn’s disease, multiple sclerosis, rheumatoid arthritis, type 1 diabetes. *, the values 1.07–1.08 represent the range of odds ratios observed across different anemia phenotypes in the UK Biobank PheWAS analysis (per genetically predicted 50 mg/dL increase in Lp(a)). AD, Alzheimer’s disease; AR, aortic regurgitation; AS, aortic stenosis; AVC, aortic valve calcification; AVD, aortic valve disorders; CAVS, calcific aortic valve stenosis; CES, cardioembolic stroke; CKD, chronic kidney disease; Diamante, DIAbetes Meta-ANalysis of Trans-Ethnic association studies; DVT, deep vein thrombosis; FinnGen, Finnish Genetic Database; I^2^, heterogeneity statistic; ICH, intracerebral hemorrhage; IMID, immune-mediated inflammatory diseases; LS, lacunar stroke; MI, myocardial infarction; MHIB, Montreal Heart Institute Biobank; MR, mitral regurgitation; MVD, mitral valve disorders; NS, not significant; PE, pulmonary embolism; SAH, subarachnoid hemorrhage; SVS, small vessel stroke; T2D, type 2 diabetes; VTE, venous thromboembolism.

While MR studies provide compelling evidence for Lp(a) causality in atherosclerotic outcomes (Table 3), the evidence for non-atherosclerotic cardiovascular outcomes presents a more complex picture (Table 4). Strong evidence supports Lp(a) causality for calcific aortic valve stenosis [41, 42, 44, 51, 52, 53]. For heart failure, the MR evidence shows significant heterogeneity that appears population-dependent, with European populations showing mostly positive associations [43, 54] or null effects [45, 55], while African [43] and Chinese [48] ancestry show null effects, with meta-analysis revealing extreme heterogeneity (*I*^2^ = 97.6%) [56]. This variation may suggest that Lp(a)’s effect on heart failure may be modified by genetic background or environmental factors. Similarly, atrial fibrillation shows modest positive associations in European [43, 55, 57] but null effects in African ancestry (OR 1.06, 95% CI 0.99–1.14) [43] and protective effects in Han Chinese (OR 0.94, 95% CI 0.90–0.99) [48], highlighting striking population-specific differences.

Notably, multiple MR studies consistently show no causal relationship between Lp(a) and type 2 diabetes (OR 0.97–1.08 across studies) [41, 58, 59, 60]. This null association distinguishes Lp(a) from triglyceride-rich lipoproteins. Unlike metabolically active lipoproteins that affect insulin signaling, Lp(a) primarily drives vascular inflammation through oxidized phospholipids without affecting glucose metabolism—suggesting Lp(a)-lowering therapies will be metabolically neutral, unlike statins. MR analysis does not support a causal relationship between Lp(a) and immune-mediated inflammatory diseases [61]. Lp(a) is causally linked to both thrombosis (especially arterial) and inflammation [62]. Its prothrombotic effects are mediated by impaired fibrinolysis and increased tissue factor, while its pro-inflammatory properties are largely due to its carriage of oxidized phospholipids and stimulation of vascular immune responses. For venous thromboembolism, MR evidence has been inconsistent—studies of individual components showed null associations [41, 42], while recent combined VTE analysis found a modest increased risk [46]. The smaller and less consistent effect sizes for non-atherosclerotic outcomes may reflect that Lp(a)’s thrombotic and inflammatory effects are primarily pathogenic in the arterial circulation or require the presence of atherosclerotic plaque to manifest their full clinical impact. This mechanistic understanding supports the focus on ASCVD as the primary target for Lp(a)-lowering interventions.

### 3.3 Dual Nature: Both Biomarker and Causal Factor

The convergence of observational and genetic evidence establishes Lp(a) as both a biomarker and causal factor. This dual validation provides the scientific foundation for both current screening recommendations and the ongoing development of Lp(a)-targeted therapies [4]. As a biomarker, Lp(a) identifies high-risk individuals who benefit from aggressive management of modifiable risk factors; as a causal factor, it represents a valid therapeutic target, justifying the ongoing clinical trials of Lp(a)-lowering approaches.

Looking forward, Lp(a) is poised to play a central role in multi-biomarker approaches to cardiovascular risk assessment. Its genetic determination and lifelong stability make it an ideal complement to dynamic biomarkers such as high-sensitivity troponin, N-terminal pro-B-type natriuretic peptide, and C-reactive protein. Such integrated biomarker panels could enable more precise identification of patients with residual cardiovascular risk despite optimal management of traditional risk factors, ultimately guiding both risk stratification and the selection of targeted interventions in precision cardiovascular medicine.

## 4. From Genetic Determinism to Modifiable Target

For decades, therapeutic nihilism toward elevated Lp(a) persisted even as genetic studies confirmed causality and biomarker research quantified risk [63]. The emergence of RNA-targeted therapeutics has shattered this dogma, demonstrating that genetic risk factors need not remain pharmacologically intractable, with novel approaches achieving Lp(a) reductions exceeding 95% and transforming a previously unmodifiable genetic burden into a druggable target.

### 4.1 The Management Void and Conventional Drugs

Conventional lipid-lowering therapies demonstrate limited efficacy against Lp(a), with some paradoxically increasing levels despite their cardiovascular benefits. Table 5 (Ref. [64]) summarizes these effects across drug classes: statin [65], PCSK9 monoclonal antibody inhibitors [66, 67, 68], PCSK9 small interfering RNA (siRNA) [69], ezetimibe [70], bempedoic acid [71], fibrates [64], niacin [72], and lomitapide [73].

**Table 5.** **Lipid-lowering drugs and their effects on Lp(a)**.

Class	Example	Mechanism	Lp(a) effect	LDL-C effect	Status (2025)	Key notes
Conventional Drugs						
Statins	Atorvastatin, Rosuvastatin	HMG-CoA reductase inhibition; upregulates *LPA* gene expression	↑10–20% or NS^a^	↓30–50%	Approved	May worsen Lp(a)-related risk (reports are heterogeneous)
PCSK9 inhibitors	Evolocumab, Alirocumab	PCSK9 monoclonal antibody; enhances Lp(a) clearance via LDL receptor upregulation, reduces apo(a) production	↓25–30%	↓50–60%	Approved	Modest Lp(a) reduction; secondary benefit
PCSK9 siRNA	Inclisiran	siRNA targeting PCSK9 mRNA; reduces hepatic PCSK9 production	↓15–25%	↓45–52%	Approved	Every 6 months injection; Modest Lp(a) reduction
Ezetimibe	Ezetimibe	NPC1L1 inhibition	↓7%	↓15–20%	Approved	Clinically insignificant for Lp(a)
Bempedoic acid	Bempedoic acid	ACL inhibition	↑2.4% or NS	↓20–25%	Approved	Minimal Lp(a) impact
Fibrates	Fenofibrate	PPARα activation	NS^b^	↓5–15% or NS	Approved	Inconsistent reports^b^, primarily for TG reduction
Niacin	Niacin	Reduces hepatic VLDL secretion	↓23%	↓10–20%	Limited use	Modest Lp(a) reduction; side effects limit utility
Lomitapide	Lomitapide	MTP inhibition	↓15–19%	↓40–50%	Approved (HoFH only)	Restricted to homozygous FH
Lp(a)-Specific Drugs						
ASO	Pelacarsen [IONIS-APO(a)-LRx]	Targets *LPA* mRNA	↓80%	↓10–15% or NS	Phase 3 (complete in 2026): Lp(a)HORIZON	Every month injection
siRNA	Olpasiran (AMG890)	siRNA targeting *LPA* mRNA	↓>95% (sustained 40–50% at 1 year)	NS	Phase 3 (ends 2026): OCEAN(a)	Every 3 months injection; potent and sustained
	Lepodisiran (LY3819469)	siRNA targeting *LPA* mRNA	↓94%	NS	Phase 3 (complete in March 2029): ACCLAIM-Lp(a)	Every 6 months injection
	Zerlasiran (SLN360)	siRNA targeting *LPA* mRNA	↓90%	NS	Phase 2 (completed 2024): ALPACAR-360	Every 4–6 months injection
Small molecules	Muvalaplin	Inhibits Lp(a) particle formation	↓85.8%	NS	Phase 2 (completed 2024): KRAKEN	First oral Lp(a)-specific drug

^a^ effect varies by statin type and dose; ^b^ meta-analysis shows no significant absolute effect of fibrates on Lp(a); however, in head-to-head trials, fibrates demonstrate a relatively greater reduction compared with statins (weighted mean differences: –2.70 mg/dL (95% CI: –4.56 to –0.84)) [64]. ACL, ATP citrate lyase; ASO, antisense oligonucleotide; FH, familial hypercholesterolemia; HMG-CoA, 3-hydroxy-3-methylglutaryl coenzyme A; HoFH, homozygous familial hypercholesterolemia; MTP, microsomal triglyceride transfer protein; NPC1L1, Niemann-Pick C1-like 1; PPARα, peroxisome proliferator-activated receptor alpha; ↑, increase; ↓, decrease.

Statins exemplify this paradox, increasing Lp(a) levels by 10–20% through upregulation of *LPA* gene expression—potentially undermining their cardiovascular benefits in patients with elevated Lp(a) levels [12, 65]. This phenomenon represents a cardiologist’s dilemma: the very medications proven to reduce cardiovascular events may simultaneously elevate a potent genetic risk factor. Despite this paradox, statins remain indicated in patients with elevated Lp(a) because their cardiovascular benefits through LDL-C reduction substantially outweigh the possible modest Lp(a) increase. MR studies suggest the cardiovascular benefit from a 38.67 mg/dL LDL-C reduction (achievable with statins) is equivalent to a 100 mg/dL Lp(a) reduction [40]—far exceeding the 10–20% Lp(a) increase caused by statins. Thus, withholding statins from high Lp(a) patients would result in net cardiovascular harm.

PCSK9 inhibitors achieve modest Lp(a) reductions, though reporting varies across studies. A meta-analysis demonstrated that these agents reduce Lp(a) by a weighted mean difference of –12.55 mg/dL (95% CI: –17.05 to –8.04 mg/dL) and are associated with reduced major adverse cardiac events (MACE) (RR 0.86, 95% CI: 0.81–0.91) in patients with coronary heart disease, though the analysis did not establish whether Lp(a) reduction independently contributes to cardiovascular benefit [67]. Similarly, while the ODYSSEY OUTCOMES trial with alirocumab demonstrated 25–30% Lp(a) reduction, it did not prove this reduction significantly contributed to cardiovascular benefits, leaving the relationship correlative rather than causal [68]. In contrast, the FOURIER trial provided stronger evidence for Lp(a)’s independent contribution, showing that evolocumab reduced Lp(a) by a median of 26.9% (interquartile range: 6.2–46.7%) at 48 weeks, with a 15% cardiovascular risk reduction per 25 nmol/L decrease in Lp(a) independent of LDL-C changes [66]. Despite these benefits, PCSK9 inhibitors are still not considered a primary Lp(a)-targeted therapy due to their modest effect compared to newer RNA-targeted therapies, which achieve reductions exceeding 95%.

Inclisiran, a PCSK9 siRNA, reduces Lp(a) by 15–25% [69], similar to monoclonal antibodies evolocumab and alirocumab, likely through enhanced LDL receptor-mediated clearance. Like ODYSSEY OUTCOMES, the ORION trials have not analyzed whether this Lp(a) reduction independently contributes to cardiovascular benefit. The ongoing ORION-4 cardiovascular outcomes trial, with results expected around 2026, will determine whether inclisiran reduces MACE.

Niacin reduces Lp(a) levels via decreasing apo(a) production rate. Extended-release niacin was associated with a significant 23% reduction in Lp(a) levels in a meta-analysis [72]. The Coronary Drug Project demonstrated the benefit of niacin monotherapy for ASCVD in men [74]; however, a combination of statin and niacin did not show clinical benefit [75, 76]. The 2024 NLA guidelines explicitly recommend against its use for ASCVD risk reduction (Class III recommendation) [22].

Lipoprotein apheresis is the first Food and Drug Administration (FDA)-approved Lp(a)-lowering intervention [33, 77]. The 2024 NLA provides a Class IIa recommendation: Lipoprotein apheresis is reasonable for high-risk patients with familial hypercholesterolemia and ASCVD (coronary or peripheral arteries) whose Lp(a) level remains ≥60 mg/dL (150 nmol/L) and LDL-C ≥100 mg/dL on maximally tolerated lipid-lowering therapy [22]. While effective at acutely lowering Lp(a) through non-specific apoB particle removal, the procedure’s benefits are transient, requiring frequent repetition.

Emerging observational evidence suggests aspirin may offer benefit for individuals with elevated Lp(a), with a recent propensity-matched study demonstrating a 46% lower risk of coronary events (HR 0.54, 95% CI, 0.32–0.94) among those with Lp(a) >50 mg/dL taking aspirin, though randomized trials are needed for confirmation [78]. Overall, these modest effects pale in comparison to the unprecedented potency of emerging RNA-targeted therapies.

### 4.2 RNA-Targeted Therapeutics and Small Molecule Inhibitors

The landscape for Lp(a) management is undergoing revolutionary transformation with therapeutic approaches targeting different stages of Lp(a) production. RNA-targeted strategies, including antisense oligonucleotides (ASOs) and siRNAs, prevent apolipoprotein(a) synthesis at the hepatic level, while small molecule inhibitors like muvalaplin disrupt Lp(a) particle assembly. These approaches provide clinicians with tools to meaningfully reduce Lp(a) levels that have remained resistant to conventional lipid-lowering therapies [79, 80].

As shown in Table 5, monthly administered ASOs like pelacarsen achieved dose-dependent Lp(a) reductions of 35–80% in phase 2 trials [6], leading to the ongoing Lp(a)HORIZON phase 3 cardiovascular outcome trial [81]. The siRNA approaches demonstrated even greater potency with extended duration: olpasiran achieved >95% reduction with quarterly dosing [7, 27, 82] and is now being evaluated in the OCEAN(a) trial [83]; lepodisiran showed 94% reduction with potential twice-yearly administration [84] and entered the ACCLAIM-Lp(a) trial [85]; while zerlasiran achieved 90% reduction with 4–6 month intervals [9] and recently completed phase 2. All maintained efficacy between doses, representing a major advance over conventional therapies requiring daily or frequent administration.

The emergence of muvalaplin as the first oral Lp(a)-lowering agent represents a pivotal advance in treatment accessibility. This small molecule inhibitor achieved up to 85.8% reductions in the KRAKEN phase 2 trial [8, 86], offering daily oral therapy that could dramatically improve patient acceptance compared to injectable approaches, particularly for primary prevention populations.

Across all RNA-targeted approaches, safety profiles have been reassuring, with mild injection-site reactions as the most common adverse effects and no evidence of off-target toxicity. Importantly, these agents specifically target Lp(a) production rather than having off-target LDL-C effects, unlike PCSK9 inhibitors which affect both.

### 4.3 The Efficacy-Outcome Gap

Despite achieving exceptional biomarker reductions with RNA-targeted therapies, a critical question remains unanswered: will these dramatic biochemical changes translate to reduced cardiovascular events? This fundamental uncertainty represents the current “waiting game” in cardiovascular medicine, where remarkable pharmacological success awaits clinical validation.

The disconnect between proven Lp(a) lowering and unproven clinical outcomes reflects the complexity of cardiovascular therapeutics. History teaches caution—numerous interventions that successfully modified biomarkers failed to improve patient outcomes. Yet the genetic evidence for Lp(a) causality, combined with the magnitude of achievable reductions, provides reason for optimism.

There are three ongoing major phase 3 cardiovascular outcome trials: Lp(a)HORIZON (pelacarsen, completing 2026) [81, 87], OCEAN(a) (olpasiran, completing 2026) [83], and ACCLAIM-Lp(a) (lepodisiran, completing 2029) [85]. These trials should provide definitive evidence whether genetically-targeted Lp(a) reduction translates to cardiovascular benefit—completing the journey from genetic insight to clinical reality.

## 5. Clinical Practice Roadmap

The imminent availability of Lp(a)-lowering therapies necessitates clear guidance for current management and preparation for integrating these agents into clinical practice. This section provides a practical roadmap for managing elevated Lp(a) with currently available tools while preparing for the therapeutic revolution ahead.

### 5.1 Today: Managing Elevated Lp(a) With Available Tools

**Fig. 1.**
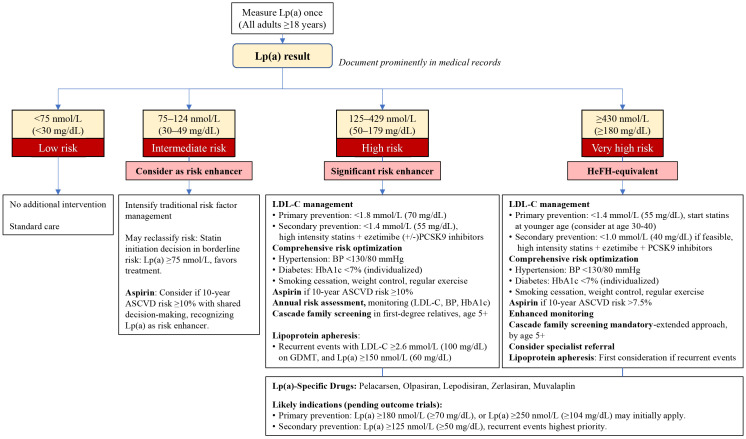
**Lipoprotein(a) management algorithm**. BP, blood pressure; HeFH, Heterozygous familial hypercholesterolemia; GDMT, guideline-directed medical therapy; HbA1c, hemoglobin A1c.

A practical treatment algorithm for Lp(a) management, based on current evidence and guidelines, is presented in Fig. 1. Universal screening is recommended for all adults ≥18 years, at least once in their lifetime. The optimal timing is 40–50 years, which balances early detection with the cardiovascular risk period. If resources are limited, priority testing would be for personal history of premature ASCVD (<55 years men, <65 years women), family history of premature ASCVD or elevated Lp(a), familial hypercholesterolemia, recurrent cardiovascular events despite optimal LDL-C control, calcific aortic valve stenosis, and intermediate cardiovascular risk (5–20% 10-year risk) requiring reclassification.

Risk stratification based on Lp(a) levels alters several key clinical decisions: (1) cascade family screening becomes mandatory when Lp(a) levels reach 125 nmol/L or higher, necessitating testing of all first-degree relatives given the strong genetic heritability [4, 12, 33]; (2) the threshold for statin initiation shifts in patients with borderline cardiovascular risk, where Lp(a) levels of ≥75 nmol/L favor treatment initiation despite otherwise equivocal risk profiles [24]; (3) aspirin for primary prevention requires careful risk-benefit assessment, with consideration warranted when Lp(a) ≥125 nmol/L and 10-year ASCVD risk ≥10%, or when Lp(a) ≥430 nmol/L with 10-year risk >7.5% [78]; (4) the addition of PCSK9 inhibitors should be considered at lower LDL-C thresholds in patients with Lp(a) levels above 125 nmol/L, recognizing both the modest Lp(a)-lowering effect and the heightened residual risk [66]; (5) lipoprotein apheresis referral becomes appropriate for patients with Lp(a) levels of 60 mg/dL or higher who experience recurrent cardiovascular events despite guideline-directed medical therapy [22, 33, 77].

Repeated Lp(a) measurements are generally unnecessary given genetic stability [12, 22]; monitoring should focus on modifiable factors like LDL-C, blood pressure, and HbA1c. Rare exceptions for repeat testing include acute phase responses (20–30% elevation during acute myocardial infarction), pregnancy-related fluctuations, verification of extremely high values before initiating apheresis, and certain conditions affecting Lp(a) levels such as declining renal function, post-menopausal status (5–10% increase), or medication effects from niacin and PCSK9 inhibitors (15–30% reduction) [12, 18, 19, 66, 72]. While these variations rarely alter management given Lp(a)’s predominantly genetic determination, clinicians should recognize these potential confounders when interpreting serial measurements.

### 5.2 Tomorrow: Preparing for Lp(a)-Specific Therapies

Healthcare systems must prepare for the imminent availability of RNA-targeted therapies. Key preparatory steps include establishing specialized lipid clinics, training healthcare providers in Lp(a) management, developing patient registries for those with elevated levels, and creating reimbursement pathways. While phase 3 trials have enrolled patients with Lp(a) thresholds as low as 70 mg/dL (175 nmol/L) for secondary prevention (Lp(a)HORIZON) [81] and 175 nmol/L for primary prevention (ACCLAIM-Lp(a)) [85], real-world implementation will likely require more stringent criteria initially.

Based on risk-benefit considerations and cost-effectiveness, patient selection may follow a tiered approach. First priority would be secondary prevention with recurrent ASCVD events and Lp(a) ≥125 nmol/L. Second would be secondary prevention with a single event and Lp(a) ≥125 nmol/L. Third would be primary prevention for patients with Lp(a) ≥180–250 nmol/L, particularly those with additional risk enhancers. These higher thresholds reflect the balance between identifying those at the highest absolute risk while awaiting definitive outcome data [12, 22].

Implementation challenges require immediate attention. Healthcare systems should establish Lp(a) testing infrastructure now, as identifying eligible patients requires systematic screening programs that may take years to implement fully. Clinical protocols must address practical questions, including the timing of therapy initiation relative to acute events, concurrent management with existing lipid-lowering agents, and monitoring requirements during treatment. Patient education programs should also be initiated immediately, as individuals with elevated Lp(a) need an understanding of both current risk management strategies and emerging therapeutic options. Beyond these clinical considerations, economic barriers will be substantial. Cost considerations based on pricing precedents for similar RNA-targeted therapies will necessitate careful patient selection and outcomes documentation to justify expenditure. Healthcare systems must develop prior authorization processes and work with payers to establish reimbursement pathways that balance access with appropriate utilization.

The focus must shift from whether we can lower Lp(a) to how we can efficiently deliver these therapies to the millions who may benefit. Centers should develop referral networks for high-risk patients and create multidisciplinary teams combining lipidology, cardiology, and pharmacy expertise. Success will require coordinated efforts between healthcare systems, payers, and pharmaceutical companies to ensure equitable access while maintaining appropriate utilization.

## 6. Conclusions

Despite establishing Lp(a) as a causal risk factor affecting 1.5 billion people globally, screening remains below 1%, leaving millions at unrecognized cardiovascular risk. Yet the emergence of RNA-targeted therapies achieving 80–95% reductions demonstrates that genetic determinism need not mean therapeutic futility. This review addresses the current implementation gap by synthesizing evolving guidelines with standardization requirements, organizing complex Mendelian randomization evidence into clinically actionable tables, and providing a practical management algorithm for the transitional period before RNA-targeted therapies become widely available. Two priorities emerge for the field: outcome trials must confirm that biochemical efficacy translates to clinical benefit and healthcare systems must eliminate barriers to systematic screening and treatment. The transformation of Lp(a) from an immutable genetic burden to a modifiable risk factor represents a pivotal advance in cardiovascular medicine. For the one in five individuals worldwide with elevated levels, closing the gap between scientific knowledge and clinical implementation is no longer just desirable—it is imperative.
